# An Analog Filter Approach to Frequency Domain Fluorescence Spectroscopy

**DOI:** 10.1007/s10895-015-1669-z

**Published:** 2015-10-01

**Authors:** R. Trainham, M. O’Neill, I. J. McKenna

**Affiliations:** Special Technologies Laboratory, National Security Technologies, LLC, 5520 Ekwill Street, Santa Barbara, CA 93111 USA

**Keywords:** Fluorescence, Frequency domain, Fluorophore, Analog filter

## Abstract

The rate equations found in frequency domain fluorescence spectroscopy are the same as those found in electronics under analog filter theory. Laplace transform methods are a natural way to solve the equations, and the methods can provide solutions for arbitrary excitation functions. The fluorescence terms can be modelled as circuit components and cascaded with drive and detection electronics to produce a global transfer function. Electronics design tools such as SPICE can be used to model fluorescence problems. In applications, such as remote sensing, where detection electronics are operated at high gain and limited bandwidth, a global modelling of the entire system is important, since the filter terms of the drive and detection electronics affect the measured response of the fluorescence signals. The techniques described here can be used to separate signals from fast and slow fluorophores emitting into the same spectral band, and data collection can be greatly accelerated by means of a frequency comb driver waveform and appropriate signal processing of the response. The simplification of the analysis mathematics, and the ability to model the entire detection chain, make it possible to develop more compact instruments for remote sensing applications.

## Introduction

Optical detection of fluorophores is often hindered by overlapping emissions from nuisance species emitting into the same spectral band. Frequently the detection limitation is not a problem of the instrumentation, but the result of broad molecular band structure of the fluorophore of interest. For example, compounds containing the uranyl radical [1, 2] emit photons in the green, in five overlapping bands, with a total spectral bandwidth of over 3000 cm ^−1^. In this case, improvement of the spectral resolution of the detector does not improve the measurement, since much of the desired signal would be discarded with the noise. This is a situation where lifetime measurements can be useful to isolate the fluorophore of interest from the nuisance emissions. The emissions of the uranyl radical have decay lifetimes of a few hundred of microseconds, whereas most nuisance emitters are shorter lived, with lifetimes in the hundreds of nanoseconds. The different time regimes make it possible to isolate the detection of the interesting fluorophore, and this can be accomplished by modulating the source driving the fluorescence and utilizing the techniques of frequency domain spectroscopy for the detection.

The techniques of time domain and frequency domain fluorescence spectroscopy are covered thoroughly in the Principles of Fluorescence Spectroscopy by Lakowicz [3]. In that text, however, the frequency domain solutions of the differential equations for fluorescence make use of direct substitution of variables. Although the procedure is correct, the mathematics are tedious and prone to error. The method of direct substitution is difficult to scale to many coupled differential equations, and it becomes quite complicated for complex driver functions. In other publications, the Fourier transform [4], sine and cosine transforms [5], and the Laplace transform [6] techniques have been applied successfully to the frequency domain fluorescence problem. Although the multi-lifetime fluorescence problem is mathematically very similar to the filter problem in analog circuit design theory [7, 8], no publications appear to treat the fluorescence problem with this simplified analysis approach. The purpose of the present article is to illustrate that fluorescence can be treated mathematically as a discrete component in a network of electronic filters, regardless of the excitation method. Use of this methodology can make a new class of instruments possible for remote sensing applications.

In analog electronics the usual approach for solving coupled differential equations is to take Laplace transforms of the equations and work directly in the frequency domain. This allows the concept of a transfer function to emerge for the response, and the scaling of fluorescence problems to more complicated driving and response functions becomes straight forward. Considerable information about a problem can be extracted from the transfer function itself, without explicit need to apply the inverse transform back into the time domain. The variable *s* of the Laplace transform is as much an operator as it is a variable; *s* is a differentiator, and its inverse, *s*^−1^ is an integrator. This concept is essential when constructing electronic circuits as analogues to differential equations and using the calculation tools of electronics to solve those equations. The basis of network theory in electronics rests upon these techniques, and the tools of electrical engineering and network analysis can be exploited to solve problems of spectroscopy. In hardware implementations, the signals are usually sampled, so the Z transform and digital filter theory [9] may be a more appropriate approach, however, we restrict ourselves in this article to the Laplace transform approach.

Analog filter theory allows unification of the optical fluorescence response with that of the electronics of the driver and detector systems. A global transfer function can be defined to describe the entire chain. Practical and unavoidable features, such as limited detector bandwidth and resulting phase shifts, or the rounding off of waveforms from driver circuit implementations can be handled elegantly by this approach. In the following sections we discuss the analog filter theory modelling and analysis of fluorescence equations, and the use of a transfer function to extract phase and modulation index (*Mod*) without transforming the equations back to the time domain. We present solutions to a few problems involving complicated driving functions, and we present examples of using the circuit tool SPICE [10] to solve fluorescence problems.

## Theory

By defining a transfer function and using the mathematics of analog filter theory the time domain and frequency domain approaches to fluorescence are not fundamentally different. The only difference lies in the appearance of the waveforms of the response functions. The simplest rate equation leading to exponential decay is:

1 $$ \frac{dn}{dt} = -\Gamma n $$

where *n* is the population of the excited state, and Γ is the decay rate. To include the effect of non-radiative de-excitation (at least for first order kinetics), Γ is replaced by a sum of all the transition rates governing the de-population of the excited state. This modification merely changes the value of the lifetime, and does not alter the exponential character of the decay. In order to have multi-exponential decay the excited state needs to be segregated into multiple groups. Effectively, multiple excited states need to be considered. If the detection is not highly discriminating (for example, a band pass filter on a photodiode) then the total measured radiation is the sum of multiple atomic transitions, with each showing its own characteristic exponential decay. Radiative decay from multiple de-excitation paths is thus described by the following equation:

2 $$ n(t) = \sum\limits_{i} n_{i} e^{-\Gamma_{i} t} $$

This equation is appropriate for very fast pulsed, or delta function, excitation. However, if the timescale of the driving excitation is comparable to any of the lifetimes, *τ*_*i*_ = 1/Γ_*i*_, then the excitation source must be explicitly accounted for in the rate equations. This leads to a convolution of source and response functions. One way of thinking about the problem is to imagine that the excitation function is a sequence of closely spaced fast pulses, where each pulse suddenly adds an excited state population, which then begins to decay away exponentially. This reasoning leads naturally to a limiting sum, which becomes a convolution integral. If we denote the excitation function by *g*(*t*), then Eq. 2 becomes:

3 $$ n(t) = \sum\limits_{i} n_{i} \int e^{-t^{\prime}/\tau_{i}} g(t-t^{\prime}) \, \mathrm{d}t^{\prime} $$

The appearance of the convolution integral immediately suggests the use of some kind of transform technique where the convolution theorem can be used to advantage. From analog electronics we recognize Eqs. 1, 2, and 3 as low pass filters.

### Single Exponential Decay

In the time domain the rate equations governing the depopulation of the fluorescing excited states are homogeneous first order differential equations, whereas in the frequency domain they are non-homogeneous equations. For both domains, however, the equations can be written as follows:

4 $$ \sum\limits_{i} \frac{dn_{i}}{dt} + \Gamma_{i} n_{i} = g(t) $$

where the *n*_*i*_ are the excited state populations, Γ_*i*_ are the decay rates, and *g*(*t*) is the driving function. If *g*(*t*) is zero, or a delta function, then we have homogeneous equations of the time domain measurement. If *g*(*t*) is periodic then we have the non-homogeneous equations of the frequency domain.

The Laplace transform of Eq. 4 is

5 $$ \sum\limits_{i} N_{i}(s)(s + \Gamma_{i}) - n_{i_{0}} = G(s), $$

where *s* is the complex frequency variable, and $n_{i_{0}}$ represent the initial state populations, which are usually zero. The transfer function for Eq. 5 is

6 $$ F_{i}(s) = N_{i}(s)/G(s) = \frac{1}{s + \Gamma_{i}} $$

**Fig. 1:**
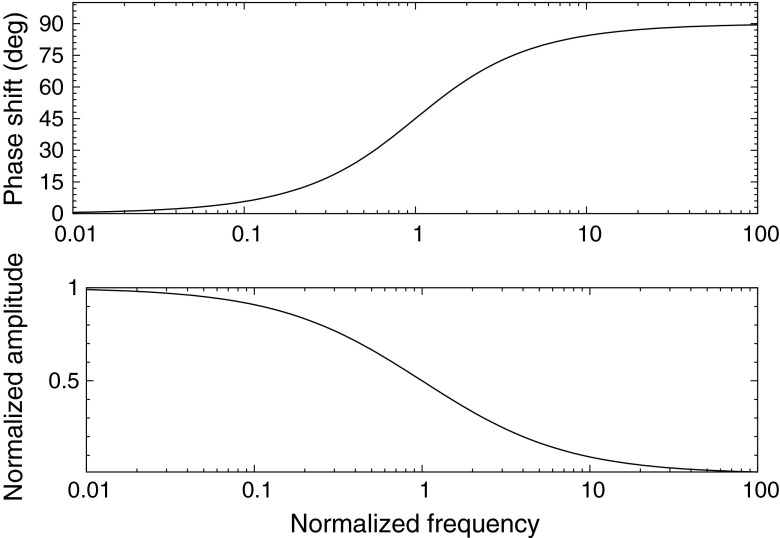
Phase shift and modulation amplitude for single lifetime exponential decay undergoing sinusoidal excitation

From this we can determine the phase shift and modulation amplitude of the filter response to a periodic driver function g(t). This is accomplished by restricting *s* to the imaginary axis, and computing the *Mod* of the transfer function for the modulation amplitude, and the ratio of the imaginary and real parts for the tangent of the phase shift. Figure 1 shows the theoretical response assuming a single lifetime emission as a function of the drive frequency *ω*. The phase shift and modulation amplitude evolve on a frequency scale determined by the reciprocal lifetime. The range of frequencies is typically two orders of magnitude centered at the angular frequency of the reciprocal lifetime *ω* = 2*π*/*τ*.

As an example of a Laplace transform solution of the decay problem, the transform Eq. 5 of the differential equation Eq. 4 with *G*(*s*)=0 for just a single *i* is

7 $$ N(s) = \frac{n_{0}}{s + \Gamma}, $$

To convert this back into the time domain we use the Bromwich integral [11] for the inverse Laplace transform

8 $$ n(t) = \frac{n_{0}}{2 \pi i} {\int}_{\gamma-i\infty}^{\gamma+i\infty} \! \frac{e^{st}}{s+\Gamma} \, \mathrm{d}s. $$

This integral is readily solved by use of the Residue theorem [12], yielding

9 $$ n(t) = n_{0} e^{-\Gamma t} $$

This is a roundabout way to solve a simple first order differential equation, but the Laplace transform technique scales readily to solve some very difficult problems. Multi-exponential decay is handled as a straight-forward sum of the various single state terms. Bandwidth limitations in the detection system, which require a convolution in the time domain, become simple products of terms in the frequency domain. Equation 7 is the transfer function for simple exponential decay of an excited atomic state. It is also the transfer function of a first order low pass filter in electronics. The norm of the complex transfer function is the modulation depth, and the ratio of the imaginary part to the real part gives the tangent of the phase shift:

10 $$ Mod \,=\, \sqrt{\frac{1}{\Gamma+i\omega}\frac{1}{\Gamma - i\omega}} \,=\, \sqrt{\frac{1}{\Gamma^{2} + \omega^{2}} }\,=\, \sqrt{\frac{\tau}{\sqrt{1+(\omega \tau)^{2}}}} $$

11 $$ tan({\Phi}) = \frac{\omega}{\Gamma} = \omega \tau $$

where Φ is the phase shift, *ω* is the angular frequency, Γ is the transition rate, and the lifetime *τ* is the reciprocal or Γ.

### Sinusoidal Driving

Another relevant example is the response of the system to a sine wave excitation function. By letting *g*(*t*)=*Asin*(*ωt*) the Laplace transform of Eq. 4 for a single decay rate is

12 $$ N(s)(s + \Gamma) - n_{0} = \mathcal{L}(A sin(\omega t)) $$

where $\mathcal {L}(sin(\omega t)$ represents the Laplace transform of the sine function. That transform is

13 $$ \mathcal{L}(A sin(\omega t)) = A \frac{\omega}{\omega^{2} + s^{2}} $$

and the expression to be transformed back into the time domain is

14 $$ N(s) = \frac{n_{0}}{s+\Gamma} + \frac{A \omega}{(s+\Gamma)(s^{2} + \omega^{2})} $$

This integral has three simple poles (i.e., singularities where the function goes to infinity), so the Bromwich integral can be evaluated by use of the Residue theorem. The solution is

15 $$ n(t) \,=\, n_{0} e^{-\Gamma t} \!+ A\left( \! \frac{\omega e^{-\Gamma t}}{\Gamma^{2} \!+ \omega^{2}}\! +\! \frac{e^{i\omega t}}{2i(\Gamma\!+i\omega)} \,-\, \frac{e^{-i\omega t}}{2i(\Gamma\!-i\omega)}\! \right). $$

The first term in Eq. 15, proportional to *n*_0_, is a simple exponential decay of any population that happens to be present at time *t* = 0. Usually, the system starts in the ground state, such that *n*_0_ = 0. The second term is a transient turn-on that damps away. It satisfies the *t* = 0 boundary condition, and it compensates for the steady state phase shift during the first few cycles of the response. The third and fourth terms in Eq. 15 are sines and cosines with phase shifts. To get a feel for them, let us look at two extremes of the driving frequency: low frequency where *ω*≪Γ, and high frequency where *ω*≫Γ. Concentrating on the steady state solution, when the transient terms have damped away, Eq. 15 becomes at low frequency (*ω*≪Γ) a sine function:

16 $$ n(t) = \frac{A}{\Gamma} sin(\omega t) $$

and at high frequency (*ω*≫Γ) Eq. 15 becomes a cosine function:

17 $$ n(t) = -\frac{A}{\omega} cos(\omega t) $$

so we see that by scanning the frequency of the drive the response transitions from a sine to a cosine, meaning that a phase shift from zero up to *π*/2 is accumulated in the response. The factor 1/Γ in front of the in-phase term means that the response is weakened by fast decay rate, and that more drive power may be required to sample fast decay fluorophores. Also, for high driving frequencies the response diminishes by 1/*ω*, meaning that the modulation depth of the response dies away to a DC offset for higher and higher frequencies.

An important difference between the atomic system and the electronics filter analog is that there cannot be a negative population in an excited atomic state. As such, the response derived for a sinusoidal driving term either must be biased positively, or the drive term must be truncated at zero, and not allowed to go negative. This is not a real limitation; it simply means that some care must be taken to assure that any results appropriated from analog filter theory are physically realistic for fluorescence. In this case, a simple offset biasing of the sine wave positive suffices, and this is precisely what is done in the laboratory.

A positively biased sine wave can be written as *g*(*t*)=*A*(1−*cos*(*ωt*))/2. Its transform into *s*-space is

18 $$ G(s) = \frac{A}{2} \left( \frac{1}{s} + \frac{s}{s^{2} + \omega^{2}}\right) $$

and the *s*-space response function is

19 $$ N(s) = \frac{A}{2} \left( \frac{1}{s} + \frac{s}{s^{2} + \omega^{2}}\right)\left( \frac{1}{s+\Gamma}\right) $$

Solving the Bromwich integral via the Residue theorem to transform back to the time domain yields

20 $$ n(t) = \frac{A}{2\Gamma} \left( 1 - \frac{\left( \frac{\omega}{\Gamma}\right)^{2} e^{-\Gamma t} - cos(\omega t) + \left( \frac{\omega}{\Gamma} \right) sin(\omega t)} {1 + \left( \frac{\omega}{\Gamma}\right)^{2}} \right). $$

**Fig. 2:**
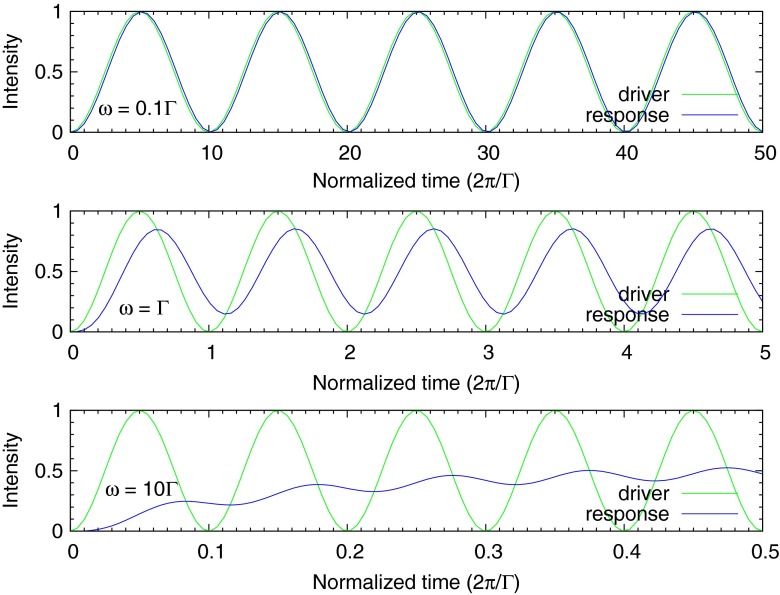
These three plots of the modelled fluorescence signal driven by a positively biased sine wave show turn-on transients, DC offsets, and decreasing modulation as the drive frequency transitions through the fluorescence decay rate. The time axis for each plot is scaled by the fluorescence lifetime 1/Γ. The plot at the *top* shows the signal for *ω* = 0.1Γ, the *middle* plot is for *ω* = Γ, and the *bottom* plot is for *ω* = 10Γ

Modeled wave forms of the driver and response functions for several frequencies are shown in Fig. 2. In the figure the time axis for each plot is scaled by the fluorescence lifetime 1/Γ. Multiplying the normalized frequency by Γ/2*π* (or equivalently, by 1/(2*πτ*)) converts the frequency scale to Hz. One sees that at low frequency the response is in phase with the driver, and increasing frequency the response lags in phase and begins to demodulate to a DC level.

### Three Level System

**Fig. 3:**
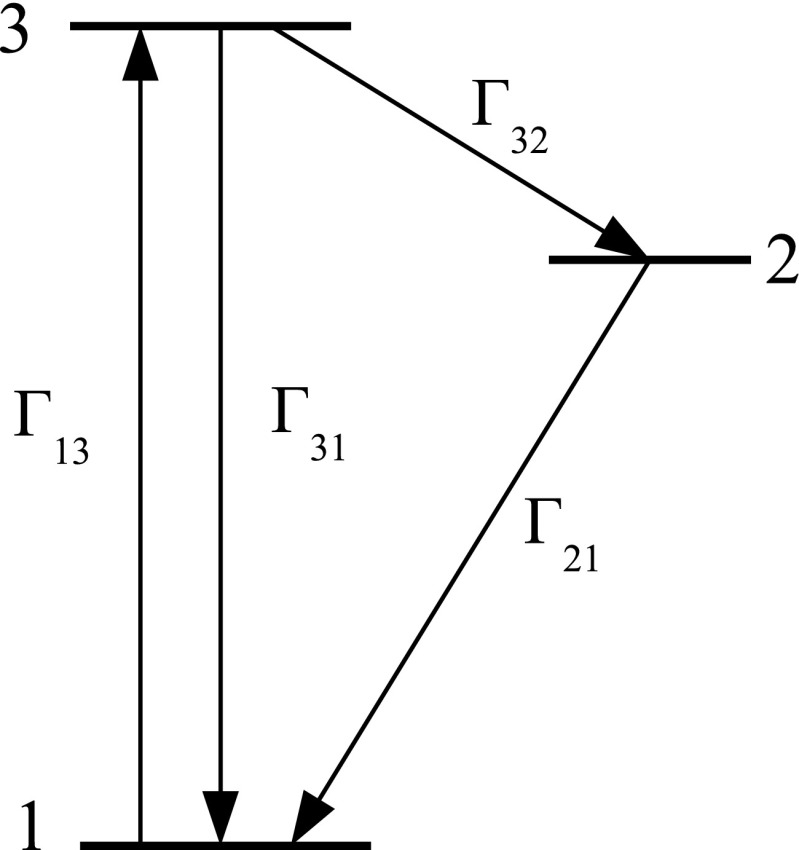
Three level system diagram. The excitation Γ_13_ pumps population from level 1 into level 3, which then rapidly decays to level 2. The fluorescence emission is directly proportional to the population of level 2

Fluorescence from a three level system can be represented by the diagram in Fig. 3. The driving term is Γ_13_, and it excites population from level 1 to level 3. Level 3 quickly relaxes to level 2. Level 2 then relaxes more slowly back to level 1. Some portion of level 3 also relaxes directly back to level 1, but that transition is typically very weak compared to the relaxation from level 3 to level 2. The fluorescence signal is proportional to the population accumulated in level 2. We denote that time dependent population by *n*_2_(*t*), and correspondingly similar notation for the populations of the other levels. The rate equations for the three levels are:

21 $$\begin{array}{@{}rcl@{}} \frac{dn_{1}}{dt} &=& \Gamma_{31}n_{3} + \Gamma_{21}n_{2} - \Gamma_{13}n_{1} \end{array} $$

22 $$\begin{array}{@{}rcl@{}} \frac{dn_{2}}{dt} &=& \Gamma_{32}n_{3} - \Gamma_{21}n_{2} \end{array} $$

23 $$\begin{array}{@{}rcl@{}} \frac{dn_{3}}{dt} &=& -\Gamma_{31}n_{3} - \Gamma_{32}n_{3} + \Gamma_{13}n_{1}. \end{array} $$

The driving term Γ_13_ can be either a delta function for a pulsed time domain measurement, or a continuous wave form for a frequency domain measurement. If we assume that the pumping does not significantly deplete level 1 then we can readily solve for the transfer function for *N*_2_ in terms of *N*_1_. The Laplace transform solution for *N*_2_(*s*) is

24 $$ N_{2}(s) = \frac{n_{2_{0}}}{s+\Gamma_{21}} + \frac{\Gamma_{32}\left( n_{3_{0}}+\Gamma_{13}N_{1}\right)} {\left( s+\Gamma_{21}\right)\left( s+\Gamma_{31}+\Gamma_{32}\right)}. $$

The terms $n_{2_{0}}$ and $n_{3_{0}}$ are initial state populations for the levels 2 and 3. They are transients which die off exponentially, and those terms are usually equal to zero. If, however, the frequency of the driver is stepped discontinuously between data samplings then whatever steady state population is left from the previous frequency step evolves according to the transient terms (which are first order lowpass filters) to the next steady state solution.

**Fig. 4:**
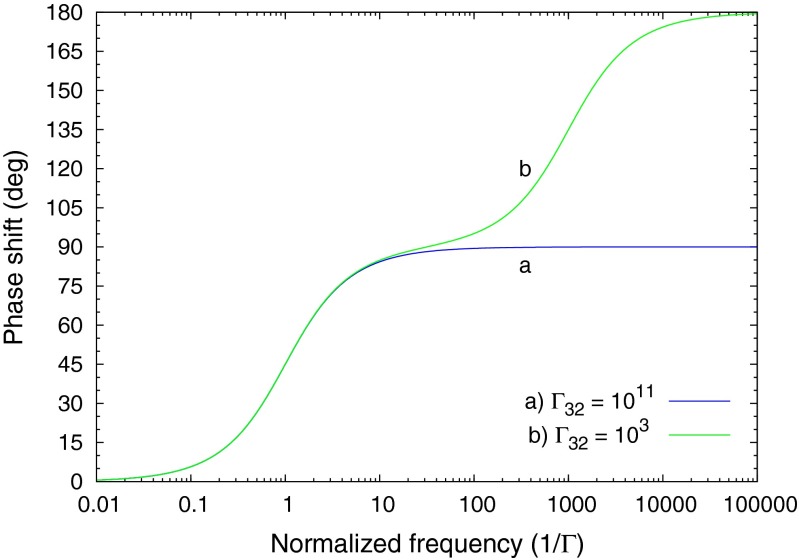
Phase shift for the 3 level system with two sets of relaxation constants

Since Γ_13_ is the driver term we factor it out to arrive at a transfer function for the three level system. The transient terms are not part of the transfer function, since they are independent of the driver function. Figure 4 shows the modelled frequency responses for two sets of relaxation parameters. Curve *a* is the response for a system with a very fast relaxation from level 3 to 2 (Γ_32_≫Γ_21_), and curve *b* is for comparable relaxation rates from 3 to 2 and 2 to 1 (Γ_32_≈Γ_21_). The second pole at -(Γ_32_+Γ_31_) in Eq. 24 lies beyond the range of relevant frequency scanning for a measurement, thus the wing of the pole approximately cancels the Γ_32_ in the numerator (assuming that Γ_32_≫Γ_31_). This is the same response as the simple lowpass filter seen previously.

Curve *b* in Fig. 4 is for the situation where the relaxation from level 3 to 2 is comparable to that from 2 to 1. The transfer function is second order, and can be represented by two cascaded lowpass filters. The rolloff in frequency of the *Mod* is steeper, and the phase shift can extend up to 180 ^∘^, since the phase shifts of the two stages are cumulative.

### Multiple Decay Lifetimes

Multi-exponential decay can be modelled as two or more excited state decays, where the photons from each state decay are undiscriminated by the detection system. The transfer function for such a process is simply a weighted sum of the individual transfer functions accounting for each excited state. Thus Eq. 6 becomes

25 $$ F(s) = \sum\limits_{k} \frac{f_{k}}{s+\Gamma_{k}}. $$

where *f*_*k*_ is the weight of each component of the excited states.

Setting *s* to *iω*, inverting Γ_*k*_ to *τ*_*k*_, and absorbing the extra *τ*_*k*_ of the numerator into the *f*_*k*_ we arrive at:

26 $$ F(i\omega) = \sum\limits_{k} \frac{f_{k}}{(1 + i\omega\tau_{k})} $$

**Fig. 5:**
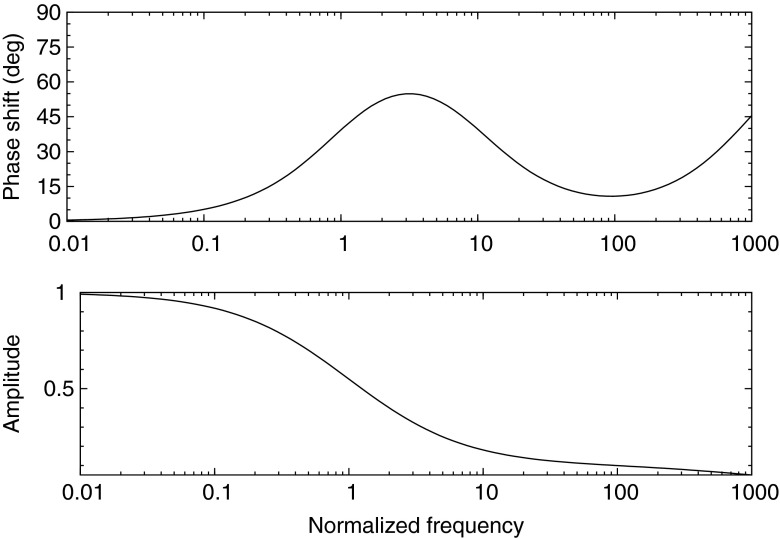
Phase shift and modulation amplitude for double exponential decay undergoing sinusoidal excitation. The two decay rates used here are *Γ*_1_ = 1 for 90 % of the signal amplitude, and *Γ*_2_ = 1000 for 10 *%* of the signal amplitude

Figure 5 shows the phase shift and modulation amplitude of the response for a double exponential decay with sinusoidal driving. The two decay rates are Γ_1_ = 1 (*τ*_1_ = 1), and Γ_2_ = 1000 (*τ*_2_ = 0.001). The frequency scale is normalized to units of the transition rate. The amplitudes have been set so that at very low frequency the amplitude of the slow decay is 0.9, and the amplitude of the fast decay is 0.1. One sees that the phase shift peaks at a value less than 90^∘^ and then turns over. This occurs because the slow component dominates the response at low frequency, but for higher frequencies the magnitude of the modulation of the slow component dies away, leaving only the fast component to determine the response, and the phase shift. The *Mod* differs subtly from that of the single exponential case, and it could be difficult to distinguish experimentally.

**Fig. 6:**
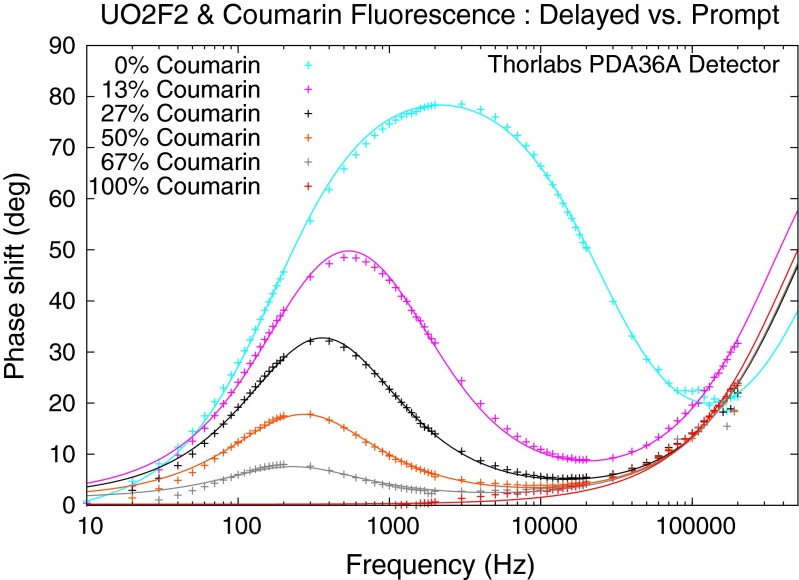
This family of curves for mixtures of fast and slow fluorescence lifetimes was generated from a uranyl fluoride sample and coumarin dye excited by a 365 nm laser diode array, and the fluorescence light collected by a photodiode and measured with a lock-in detector

The behavior of the response phase shift with drive frequency is the most dramatic effect of multiple decay lifetimes. Figure 6 shows a family of experimental curves for known mixtures of fast and slow fluorophores. The two samples used were coumarin dye, with a picosecond lifetime, and uranyl fluoride, with a half millisecond lifetime. Varying the mixture of the two samples via *f*_*k*_ reveals that the fast lifetime fluorescence partially masks the full phase shift of the slow component. This is because the amplitude of the slow component diminishes as it is driven faster than its natural decay rate. Its fluorescence becomes a DC light level. The fast component, however, still oscillates in phase with the driver at full amplitude, so its signal tends to dominate the response as the drive frequency is increased. These experimental results were collected using a Nichia NCSU033AE LED array to provide excitation light at 365 nm. The signal waveform to drive the LED array was generated by an Agilent 33250A function generator and boosted by a Tegam 2348 power amplifier. The fluorescence light was filtered by an Andover 550FS40-50 bandpass filter to remove the driver light, and the photodiode detector was a Thorlab PDA36A set at 0 dB gain. The signal was measured by an EG&G Signal Recovery 7260 lock-in detector.

### Multiple Frequency Driving Functions

#### Square Wave

Driving fluorescence with a square wave excitation has several advantages over sine wave driving. The circuitry for square wave driving a laser diode is a simple switching circuit, and the non-linear response of the diode for rapid turn-on and turn-off is not an issue. For sine wave driving, however, maintaining waveform fidelity close to laser threshold is quite tricky, and this requires more elaborate circuitry with feedback. Another advantage of the square wave is that the fundamental term in the harmonic series of the square wave is 27 % more intense than that of a single frequency sine operating from the same power supply. Finally, a square wave drive allows multiple frequencies to be sampled simultaneously, and this feature can be exploited to perform faster data collections.

The Laplace transform for a positively biased square wave multiplied by the fluorescence low-pass filter transfer function is

27 $$ R(s) = F(s)G(s) = \frac{1}{s+\Gamma} \left( \frac{1}{s} + \frac{1 - e^{-s\pi/\omega}}{s\left( 1+e^{-s\pi/\omega}\right)}\right) $$

where *ω* is the square wave’s angular frequency. The first term in the parentheses is the positive bias, and it produces an asymptotic exponential drift to the DC offset. The other term in the parenthes is when multiplied by the filter transfer function produces both oscillatory and transient terms. It has three terms where poles occur. One is at the origin, but it is cancelled by a zero at the origin. Another is at −Γ, and this produces a transient turn-on. Finally, an infinite set of poles occur at locations along the imaginary axis wherever the exponential term in the denominator is equal to -1. Those poles occur where

28 $$ e^{s\pi/\omega} = e^{ik\pi} $$

for k odd. Since the complex variable *s* is located in an exponential, the poles of the expression should be evaluated with help from L‘Hospital’s rule. For the infinite set of poles along the imaginary axis, the exponential term introduces a scaling factor of *kω*/*π* in the denominator at each pole. Since *k* is odd the oscillatory terms consist of odd harmonics weighted by the order of the harmonic. Transforming back to the time domain produces the following function.

29 $$ r(t) \,=\, \frac{1}{\Gamma}\left( \!1\,-\,\frac{e^{-\Gamma t}}{1\!+e^{\pi\Gamma/\omega}}\!\right) + \frac{2}{\pi} \sum\limits_{k=odd}^{\infty} \frac{\frac{\Gamma}{k} sin(k \omega t) \,-\, \omega cos(k \omega t)}{\left( k^{2} \omega^{2} \,+\, \Gamma^{2} \right)} $$

**Fig. 7:**
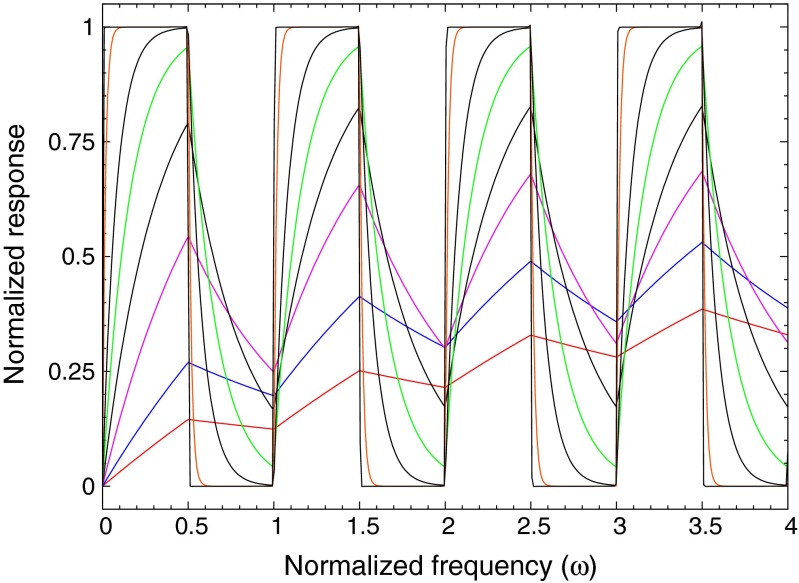
The fluorescence response to a square wave drive signal has a shark’s fin appearance at frequencies comparable to the decay rate. The family of curves shown here is for response wave forms ranging in frequency from *ω* = 0.01Γ to *ω* = 10Γ

When the square wave drive frequency *ω* is slow compared to the fluorescence decay rate Γ the fluorescence response is a rounded square wave. When the frequency is comparable to the decay rate then the response has a shark’s fin appearance. At higher frequencies the response becomes a triangle wave with smaller and smaller amplitude. At higher drive frequencies the turn-on transient persists over multiple cycles before settling down and oscillating about a constant DC offset. Examples of the response wave forms for drive frequencies ranging from *ω* = 0.01Γ to *ω* = 10Γ are shown in Fig. 7.

**Fig. 8:**
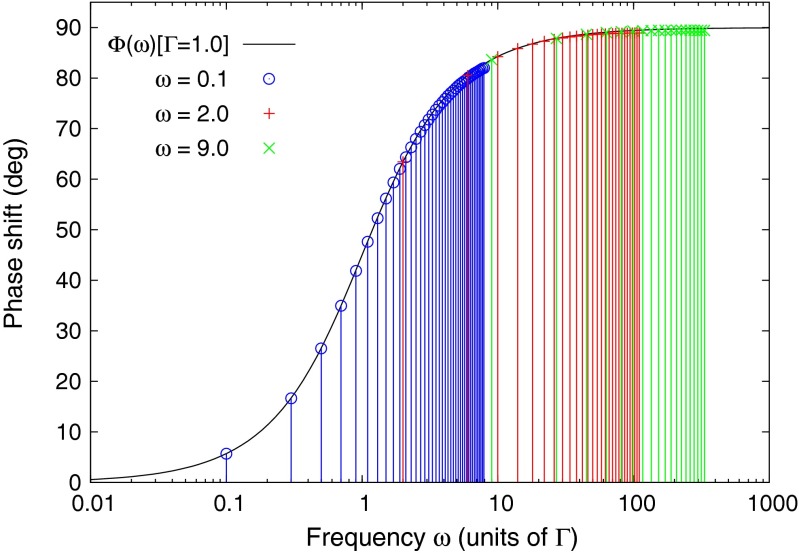
The modelled phase of the fluorescence response is reproduced using only three frequency settings for the driver excitation: *ω* = 0.2, *ω* = 1, and *ω* = 9, where *ω* is in units of the fluorescence decay rate Γ

The harmonic content of a square wave can be exploited to sample the fluorescence response over an extended frequency range using only a few frequency steps of the driving term. In Fig. 8 the modelled phase response has been reproduced with only three frequency settings for the driver: *ω* = 0.2, *ω* = 1, and *ω* = 9, where *ω* is in units of the fluorescence decay rate Γ.

**Fig. 9:**
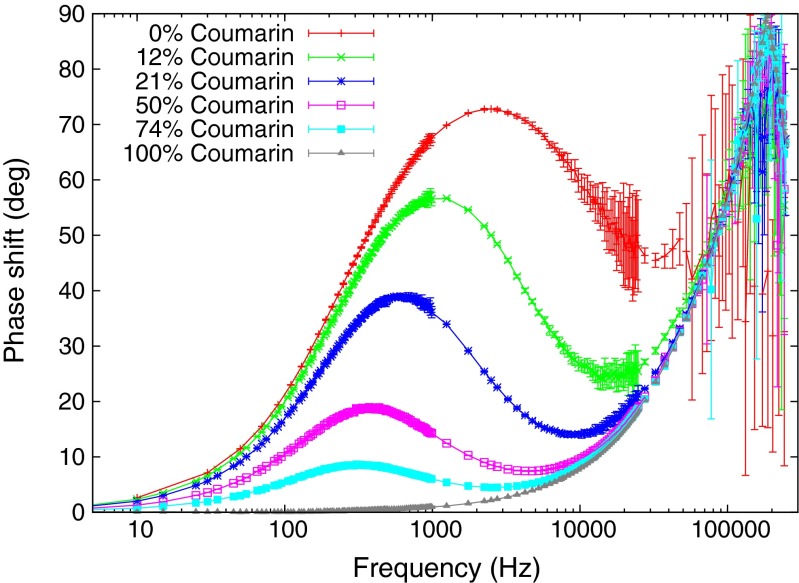
This family of curves was generated using the same uranyl fluoride and coumarin fluorophores, the same laser, and the same photodiode as were used for Fig. 6, These curves were constructed using only three drive frequencies: 10 Hz, 250 Hz, and 2.5 kHz. The driver signal in this measurement was a square wave, and the data were collected by a fast digitizer. The data were processed for phase responses of the harmonic components up to 50 harmonics

Figure 9 shows experimental results from data collected with only three drive frequencies of the square wave, and exploitation of the phase shifts of harmonics up to harmonic 50. By comparison, the data in Fig. 6 were collected using a pure sine wave and 80 frequency samples for each curve. The experimental setup for Fig. 9 was identical to the setup used to collect the data shown in Fig. 6, except that the lock-in detector was replaced by a National Instruments USB-6251 fast waveform digitizer. The time domain signals from the driver and response waveforms were each averaged over a fixed number of cycles, typically 100, and then transformed into the frequency domain by Fast Fourier Transform (FFT). The FFT arrays were then averaged for mean and standard deviations of all complex frequency values of the harmonics. The phase delays between the response and driver signals of harmonics up to 50 were computed, and these are shown in Fig. 9. The error bars in the plot are the standard deviations of the computed phase shifts. The three frequencies used to reconstruct the family of phase shift curves were 10 Hz, 250 Hz, and 2.5 kHz. The various curves in the figure are for different mixtures of fast and slow lifetime fluorophores. The data collection time for each curve in Fig. 9, using three drive frequencies, was only a few seconds. By contrast, the data collection time in Fig. 6 required 6 minutes for each curve.

#### Frequency Comb

The square wave has harmonic intensities proportional to the inverse of the harmonic order. Consequently, the power of the harmonics weakens rapidly as the harmonics probe higher frequencies. This situation is not desirable, since the *Mod* of the fluorescence response also falls off at higher frequency. Ideally, the driver’s harmonic terms would have uniform intensity, or perhaps increasing intensities with harmonic order. An arbitrary waveform generator can synthesize any desired harmonic content up to the instrument’s bandwidth limit, but the problem of linearity of the excitation laser’s response would compromise performance. Also, incorporating an arbitrary waveform generator into an apparatus would add to the cost and complexity of the instrument.

A better alternative to an arbitrary waveform excitation driver is actually a rather simple solution. It is the Shah function, or the Dirac Comb. These wave forms are simple pulse trains. The Dirac comb is infinitely thin and intense, but it integrates to a finite value, and the Shah function is a discrete domain realization of the Dirac comb. The harmonic content of the Dirac comb has all harmonic orders of equal intensity, with all orders in phase with each other, thus the Dirac comb can also be called a frequency comb, or a phase comb. The experimental realization of the frequency comb is simply a short pulse laser with an adjustable repetition rate. The repetition rate is the fundamental frequency of the driver, and since the pulses are sharp, many harmonic orders of that repetition rate are present in the signal. In the limit that the pulse train is infinitely long and the individual pulses infinitely sharp the frequency comb contains an infinite number of harmonic orders.

A realization of the frequency comb can be approached by considering a periodic rectangle wave. If we let the duration of an individual pulse be *T*_1_, and the pulse train repetition rate be *T*, then the Laplace transform of a single rectangular pulse is

30 $$ F_{p}(s) = {\int}_{0}^{T_{1}} \! e^{-st} \, \mathrm{d}t = \frac{1-e^{-sT_{1}}}{s}, $$

and the transform of the pulse train is

31 $$ F_{_{T}}(s) = \sum\limits_{k=0}^{\infty} \frac{1-e^{-sT_{1}}}{s} e^{-sTk} = \frac{1-e^{-sT_{1}}}{s(1-e^{-sT})} $$

If we next define the duty cycle of the pulse train to be *D* = *T*_1_/*T* then the transfer function becomes

32 $$ F_{_{T}}(s) = \frac{1-e^{-sTD}}{s(1-e^{-sT})}. $$

This transfer function has poles along the imaginary axis at *s* = *i*2*πk*/*T*, where *k* is an integer representing the harmonic order. The Residue theorem and the Bromwich integral have factors of *i*2*π* that cancel, so the Residue at each pole is

33 $$ Res = \frac{1-e^{i2{\pi}kD}}{i2{\pi}k}. $$

The *Mod* of the transfer function at each pole is therefore

34 $$ Mod = D \sqrt{\left( \frac{sin({\pi}kD)}{{\pi}kD}\right)^{2}}, $$

and the phase shift is

35 $$ \Phi = {\pi}kD. $$

The *Mod* of the transfer function of the pulse train is a frequency comb with an envelope function of *sin*(*x*)/*x*, where *x* = *πkD*. Wherever *kD* is integer valued the harmonic peak has zero amplitude. The phase shift for each harmonic peak is linear in the harmonic order with a slope of *πD*. In the special case of a square wave, where the duty cycle is *D* = 1/2, all even harmonics are null, and all of the phase shifts are identical, modulo *π*/2. For other duty cycle values, a harmonic peak is nulled whenever *kD* becomes an integer.

**Fig. 10:**
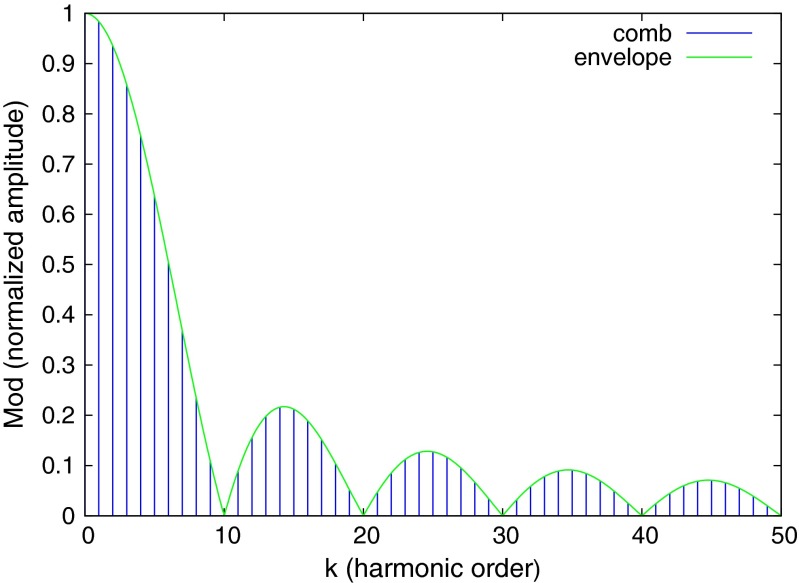
The *Mod*, or the normalized power spectrum, for a 10 % duty cycle pulse train has nulls at every tenth harmonic. The amplitudes of the harmonic peaks follow an envelope function of *abs*(*sin*(*x*)/*x*), with *x* = *πkD*, where *k* is the harmonic order and *D* = 1/10 is the duty cycle of the pulse train

**Fig. 11:**
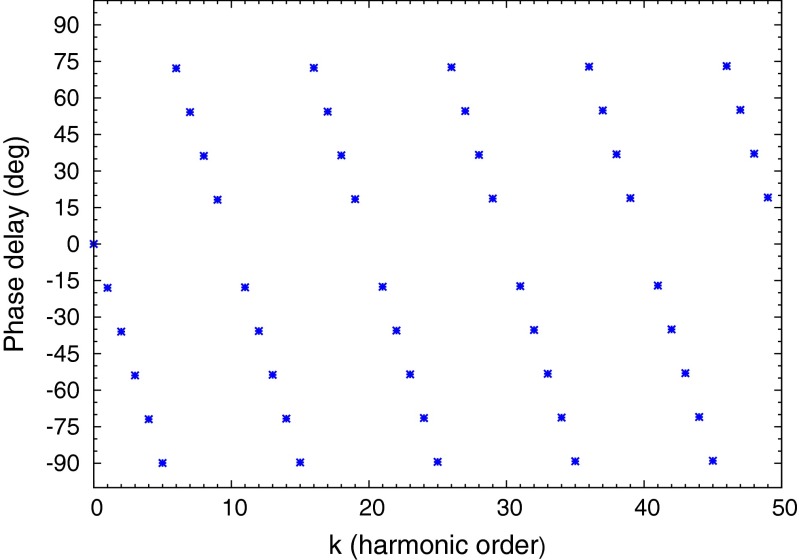
The phase delays for the harmonic peaks of a 10 % duty cycle pulse train decrement linearly with a slope of 18 ^∘^ (*π*/10), and a null occurs at every tenth harmonic. The sawtooth appearance of the graph is due to the convention of mapping phase shifts into the range [−*π*/2, *π*/2] to be consistent with the arc tangent function

For a duty cycle of *D* = 1/10, the harmonic peaks lie under the *sin*(*x*)/*x* envelope, where *x* = *πk*/10, and every tenth harmonic peak is null. This is illustrated in Fig. 10. The amplitudes of the harmonic peaks remain significant out to high order, thus the frequency comb allows many sample points of the fluorescence response function to be measured simultaneously with a single drive frequency. The phase of each harmonic is linearly delayed with the harmonic order. Figure 11 shows the phase delays versus harmonic order for a pulse train with a duty cycle of 1/10. One sees that every 10’th harmonic is missing, and the phase shifts of the harmonic peaks are Φ_*k*_ = *kπ*/10. The sawtooth appearance of the phase shifts in Fig. 11 is due to the modulo ±*π*/2 convention of representing phase shifts of periodic signals.

### Circuit Representations

Perhaps the most important heuristic to be found in the similarity of the fluorescence problem to analog filter theory is the ability to use the tools of electronics to solve the fluorescence equations. After moderate experience in circuit analysis one can draw circuits of most arbitrary transfer functions simply by inspection. The key concepts are that poles are represented by integrators, and zeros by differentiators. The transfer function can be factored and rearranged algebraically in several ways, and sometimes it is convenient to convert a zero acting on an input into a pole acting on the output and feed it back into the circuit. Usually, several different circuit realizations can be made to represent a single transfer function.

Once a transfer function has been modelled as a circuit then SPICE [10] can generate phase plots and Bode plots (which are plots of the *Mod*). Transient and steady state responses to arbitrary driving functions can be solved easily, and the analysis of the circuit can verify a fluorescence solution obtained analytically. Insights into issues of adequate sampling and of responses to exotic and complicated driving functions can be obtained from a SPICE analysis.

The transfer function of Eq. 24 can be represented by the following circuit: the initial values of $n_{2_{0}}$ and $n_{3_{0}}$ have been set to zero, but they could easily have non-zero starting voltages. The driving term Γ_13_ is the voltage source, and the factored terms in the denominator of Eq. 24 are cascaded low pass filters. Since each low-pass can introduce a phase shift up to 90 ^∘^ the cascade of two of low-pass filters can phase shift the input by up to 180 ^∘^.

**Fig. 12:**
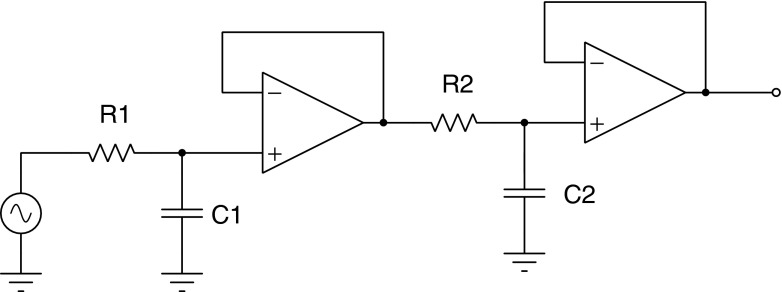
This circuit is a low pass filter realization of the Laplace Transfer function for the three level fluorescence of Eq. 24, where $n_{2_{0}}=n_{3_{0}}=0$

**Fig. 13:**
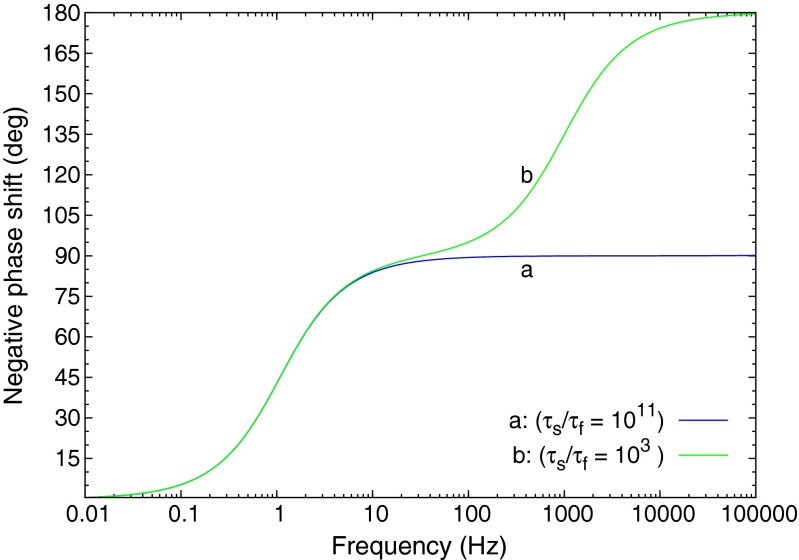
The AC transfer characteristic of the SPICE implementation Tina-TI SPICE was used to generate the phase shift plotted here. These phase shifts correspond to two sets of fast and slow *RC* time constants for the double low pass circuit shown in Fig. 12. These results are identical to that of the calculated Laplace solutions shown in Fig. 4. The 2*πRC* lifetime of slow component (stage 1 of the circuit) for the both SPICE calculations is *τ*_*S*_ = 1. Curve **a** above shows the phase shift for *γ*_*F*_ = 1/*τ*_*F*_ = 10^11^, and curve **b** for *γ*_*F*_ = 1/*τ*_*F*_ = 10^3^

Figure 13 shows the phase response calculated by Tina-TI SPICE [13] of the circuit in Fig. 12 for two sets of *RC* time constants of the cascaded low pass filters. The curve labeled *a* is for a very fast relaxation from level 2 to 3 (refer to Fig. 3), and this response is for the usual situation found in fluorescence. Curve *b* in Fig. 13 is for a slower relaxation from level 2 to 3, and one sees that the phase shift of the response can extend beyond 90 ^∘^ when the driving frequency reaches the vicinity of the level 2-to-3 relaxation transition rate. For both curves the 2*πRC* lifetime of the first stage (slow component) was set at 1 second. The electronic component values were *R*_1_ = 159*k*Ω and *C*_1_ = 1*μF*. For curve a) the second stage electronic components values were *R*_2_ = 1.6*k*Ω and *C*_2_ = 100*nF*. For curve b) the second stage component values were *R*_2_ = 0.159Ω and *C*_2_ = 10*pF*.

The circuit in Fig. 12 was drawn by inspection of the transfer function of Eq. 24, but it could been drawn directly from the three level diagram in Fig. 3 without having to solve the algebraic equations in Laplace space. In the electronic analogue each level population of Fig. 3 represents a voltage, and the transition rates represent either drive terms Γ_13_, or filter terms, Γ_31_, Γ_23_, Γ_21_. Each transition term of Fig. 3 is a circuit element joined to the other circuit elements in the figure. The correspondence is quite useful and powerful. Even if a hypothetical fluorescence system appears to be too onerous to solve in Laplace space then it should still be possible to draw the electronic equivalent and solve the problem with SPICE. Certain simplifying assumptions, such as negligible initial state depletion, are not necessary. SPICE can calculate the response behavior for arbitrary initial conditions, even when the system is driven hard.

**Fig. 14:**
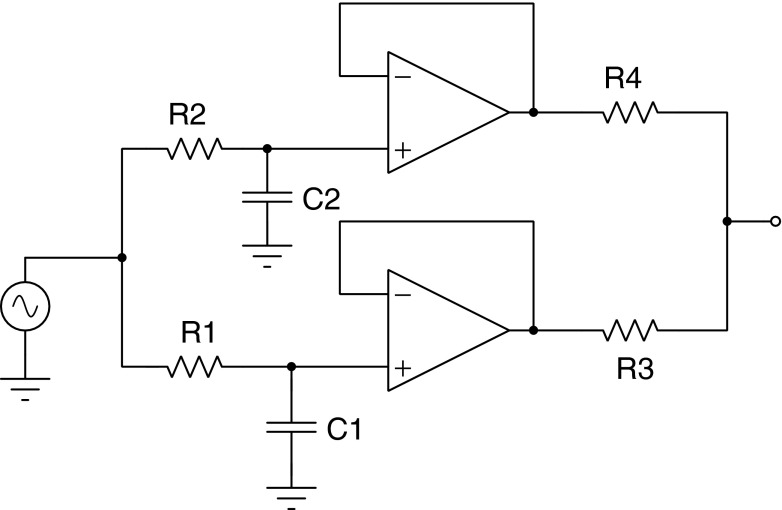
This circuit is a realization of the transfer function for the two lifetime fluorescence of Eq. 26

**Fig. 15:**
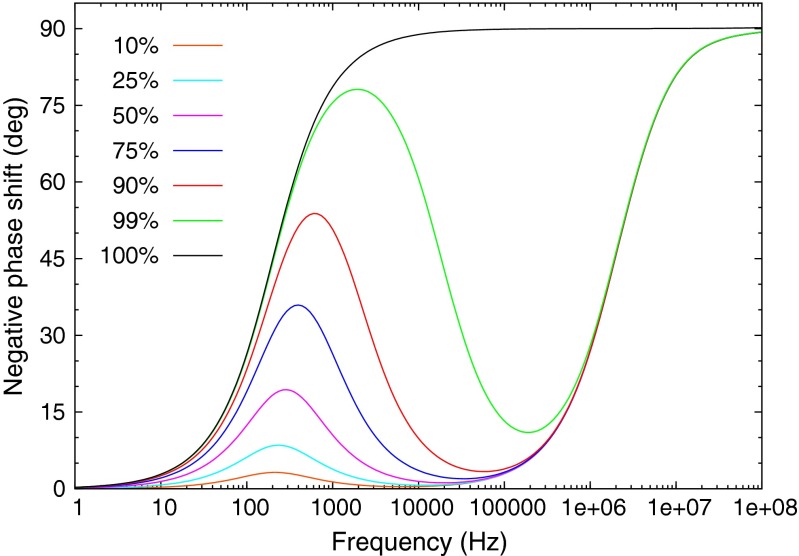
This family of curves of phase shifts for different mixtures of two lifetimes was calculated by the Tina-TI SPICE. The slow and fast decay rates are separated by 4 decades, and the actual frequencies used in the simulation were 100 Hz and 1 MHz. However, the frequencies can be scaled appropriately. The ratios of intensity of the slow component to the fast component ranges from 10 to 100 %. This figure should be compared to Figs. 6 and 9

The equivalent circuit for the transfer function with two lifetime components of Eq. 26 is shown in Fig. 14. This circuit was loaded into Tina-TI SPICE using two fixed *RC* time constants for slow and fast components. In SPICE different summing resistor values were substituted for R3 and R4 in order to generate the family of mixture curves shown in Fig. 15. The actual frequency values used for the slow and fast transition rates in the circuit were 100 Hz and 1 MHz, but those frequencies can be rescaled arbitrarily to represent other decay rate regimes. The four decades difference between the two decay rates scales appropriately, so the simulation could equally apply to a problem with GHz decay rates.

For both the low-pass circuit and the fluorescence response the phase shift is, in fact, a phase lag. In electronics it is customary for a phase lag to be negative. The plots here show the phase lag as a positive phase shift, which is the convention in the literature on frequency domain fluorescence.

One caveat of using a circuit to model a fluorescence problem is that SPICE may calculate more detail than would appear in the circuit taken at face value. The op-amp models in SPICE have varying degrees of realistic behavior, and at certain frequencies the solution may be dominated by the filter characteristics of the op-amp model itself, rather than by the other circuit components. For example op-amp models in Tina-TI SPICE includes a selectable slew rate, realistic impedances, and two poles for phase responses. One must be mindful of these features when converting a transfer function into a circuit. It is prudent to choose an appropriate scaled frequency range and to move the poles of the op-amp into regions where they do not affect the expected response.

## Conclusion

Frequency domain fluorescence measurements in atomic and molecular physics can be modelled as electronic analog low-pass filters and processed with the tools of electrical engineering. The mathematical equivalence of fluorescence to analog filters permits a unified treatment of the entire fluorescence chain by cascading transfer functions. Driver terms, fluorescence response, and detection electronics can all be modelled on equal footing. Phase shifts and amplitude distortion from high gain and bandwidth limited detection electronics can be readily accounted for, and complicated fluorescence rate equations in both time and frequency domains can be handled elegantly. Equivalent circuits can be constructed to model fluorescence equations, and tools such as SPICE can be used to generate Bode plots, phase plots, and transient response signals. Frequency combs permit the sampling of many response points with just a few driver frequencies, so data collection can be accelerated considerably by use of driver functions rich in harmonic structure. This is advantageous in situations where measurement dwell times are limited. These techniques of modelling and analysis make it possible to design more compact instrumentation for remote sensing applications.
